# Fatal Liver and Lung Alveolar Echinococcosis with Newly Developed Neurologic Symptoms due to the Brain Involvement

**DOI:** 10.1055/s-0037-1603502

**Published:** 2017-05-25

**Authors:** Ahmet Tuncay Turgut, Mehmet Turgut

**Affiliations:** 1 Department of Radiology, Hacettepe University Faculty of Medicine, Ankara, Turkey; 2 Department of Neurosurgery, Adnan Menderes University Faculty of Medicine, Aydın, Turkey

Dear Sir,

We read with great interest the article by Kvascevicius et al entitled “Fatal liver and lung alveolar echinococcosis with newly developed neurologic symptoms due to the brain involvement.” 1 As stated by the authors, alveolar echinococcosis (AE) caused by *Echinococcus multilocularis* , a cyclophyllid tapeworm, is a disease in certain terrestrial mammals, including wolves, foxes, jackals, coyotes, domestic dogs, and humans. 2 3 Unlike *Echinococcus granulosus* , *E. multilocularis* produces many small cysts within the internal organs of the infected animal. 2 3 For the sake of completeness of the information presented, there appeared to be some points that we felt obliged to comment upon:

1. Radiologically, what is most crucial for the follow-up of such a case is that the evaluation of the course of the disease should preferably be made by comparing the studies performed by the “same” imaging tool. Therefore, the comparison between preoperative magnetic resonance imaging (MRI) and postoperative computed tomography (CT) scans poses as a limitation in the current report.
2. There is no doubt that it is important to differentiate AE from cerebral malignancies, such as glial and metastatic tumors, and other infective cerebral diseases involving the cerebrum, including tuberculoma, in patients from areas endemic for hydatidosis. In spite of the fact that CT and MRI are the most accurate diagnostic tools, the presented cystic radiological appearance of the lesion in this patient is unfortunately not pathognomonic for AE.
3. Histopathologically, necrosis with granulomatous reaction composed of multinucleated giant cells and epithelioid cells and inflammatory cells surrounding multiple periodic acid-Schiff-positive cysts are key findings of AE, as seen in the surgical specimen of the current case. Nevertheless, total magnification rate in light microscopes which were not given in Fig. 5 is a significant drawback for the present report because the thoroughness of the presentation of a scientific contribution is as important as a proper content, which apparently falls to the authors of the article.
4. Finally, Schmid et al suggested that gamma knife radiosurgery may also be considered in the treatment of AE. 4 However, as clearly demonstrated in the current case, it is still a life-threatening procedure, in spite of all the advancements in neuroradiological techniques and therapeutic modalities, including surgery plus adjuvant chemotherapy.

## References

[1] Kvascevicius R Lapteva O Al Awar O Lung alveolar echinococcosis with newly developed neurologic symptoms due to the brain involvement Surg J 2016 2 e83 e88 10.1055/s-0036-1592122 PMC5553476 28824996

[2] Turgut M Hydatidosis of the Central Nervous System: Diagnosis and Treatment New York, Heidelberg, Dordrecht, London Springer 2014

[3] Turgut M Hydatidosis of central nervous system and its coverings in the pediatric and adolescent age groups in Turkey during the last century: a critical review of 137 cases Childs Nerv Syst 2002 18 12 670 683 12483350 10.1007/s00381-002-0667-z

[4] Schmid M Pendl G Samonigg H Ranner G Eustacchio S Reisinger E C Gamma knife radiosurgery and albendazole for cerebral alveolar hydatid disease Clin Infect Dis 1998 26 06 1379 1382 9636867 10.1086/516351

